# Case Report: Expanded delineation of phenotype of TRPM3-related neurodevelopmental disorders

**DOI:** 10.3389/fped.2024.1435053

**Published:** 2024-11-21

**Authors:** Agnieszka Pawelak, Artur Polczyk, Ewelina Wolańska, Magdalena Kłaniewska, Mateusz Biela, Aleksander Basiak, Maria Franaszczyk, Małgorzata Rydzanicz, Rafał Płoski, Robert Śmigiel

**Affiliations:** ^1^ Department of Genetics, Medical University of Wroclaw, Wroclaw, Poland; ^2^ Medical Education and Simulation Laboratory, University Centre of Physiotherapy and Rehabilitation, Wroclaw Medical University, Wroclaw, Poland; ^3^ Department of Family and Pediatric Nursing, Wroclaw Medical University, Wroclaw, Poland; ^4^ Department of Pediatrics, Endocrinology, Diabetology and Metabolic Diseases, Medical University of Wroclaw, Wroclaw, Poland; ^5^ Department of Medical Genetics, Medical University of Warsaw, Warsaw, Poland

**Keywords:** TRPM3, intellectual disability, neurodevelopment, autism spectrum disorder, *de novo* variants

## Abstract

The TRPM3 gene, part of the transient receptor potential (TRP) cation channel family, plays crucial roles in sensory perception and ion transport. Mutations in TRPM3 are linked to a range of neurological and developmental disorders. The c.2509G>A variant specifically leads to a substitution at position 837 in the protein, which is likely critical for its normal function. This study presents a male pediatric patient with a pathogenic TRPM3 variant c.2509G>A [p.(Val837Met)], contributing to a complex clinical phenotype characterized by developmental delays, significant hypotonia, and neurological abnormalities. The patient demonstrated delayed motor milestones, including the inability to sit independently until 20 months, and abnormal EEG findings without epileptic seizures. Ophthalmologic issues, such as hyperopia and astigmatism, were also identified. Behavioral abnormalities and cognitive impairment aligned with previous reports of TRPM3-related neurodevelopmental disorders. This case highlights the phenotypic variability linked to the p.(Val837Met) variant and emphasizes the need for further research into effective therapeutic strategies for TRPM3-associated conditions.

## 1 Introduction

The TRPM3 (transient receptor potential melastatin 3) gene encodes a calcium-permeable ion channel, a member of the TRP family, widely expressed in various tissues, including the nervous system and pancreatic beta cells (1–4). Mutations in TRPM3 have been linked to significant neurological phenotypes, with the p.(Val837Met) variant emerging as one of the most studied pathogenic mutations (5–9). This mutation induces profound changes in channel gating, resulting in increased basal activity, altered sensitivity to heat, and enhanced ligand reactivity (5, 10). These gain-of-function properties are key to understanding the pathogenic mechanisms underlying the observed clinical manifestations, such as epilepsy, developmental delays, and neurodevelopmental disorders.

It has been demonstrated that TRPM3 gene variants result in significant alterations in channel gating at the cellular level, including increased basal activity, heightened heat sensitivity, and altered responses to ligand modulation. Such a pronounced gain of channel function underlies epileptic activity and neurodevelopmental symptoms (5). All known disease-associated TRPM3 variants exhibit gain-of-function characteristics, though the specific functional impact varies depending on the mutation. The p.(Val837Met) variant, in particular, has been shown to maintain relatively normal thermal sensitivity, while its heightened basal and ligand-induced activity distinguishes it from other variants (5, 10). This variant leads to both neurological symptoms and ocular manifestations, including hyperopia and astigmatism, in comparison to other variants correlated with the occurrence of glaucoma and cataract. This suggests a broader systemic impact of TRPM3 dysfunction (4, 10–12).

Emerging studies suggest that the clinical phenotypes observed in individuals with TRPM3 mutations stem primarily from dysregulated calcium homeostasis, a direct consequence of the altered ion channel activity. The increased calcium influx through TRPM3 triggers secondary signalling cascades that affect multiple cellular processes, including neuronal excitability, synaptic transmission, and gene expression. However, the precise molecular mechanisms linking TRPM3 dysfunction to specific clinical symptoms remain an active area of investigation (1, 5, 10).

This study explores a pediatric male with a complex clinical phenotype linked to a pathogenic TRPM3 gene variant [c.2509G>A; p.(Val837Met)]. The patient presented with developmental delays, psychomotor impairments, and neurological abnormalities, with genetic testing revealing the pathogenic TRPM3 variant. Clinical manifestations encompassed hypotonia, delayed motor milestones, cognitive impairments, and behavioral abnormalities. Visual disturbances, including hyperopia and astigmatism, added to the complexity of the phenotype. This case contributes to the growing body of literature on TRPM3-related pathologies, emphasizing the gene's role in neurodevelopment and its potential as a target for future therapeutic interventions. However, there is still an open issue regarding the role of this protein in cells in which TRPM3 expression has been demonstrated.

## 2 Case report

A male newborn was born from the third pregnancy, second birth, by caesarean section (abnormal CTG recording) at term (38 week of gestation) with a body weight of 3,200 g, length 52 cm, OFC 35 cm, in good condition (assessed on the Apgar scale at 10 points). The course of pregnancy was ordinary, apart from the risk of premature birth. Prenatal ultrasound examinations showed no abnormalities. During pregnancy, the mother felt fetal movements well. No abnormalities were detected after birth. The patient was breastfed until the age of 6 months and was suckling well.

**Figure 1:**
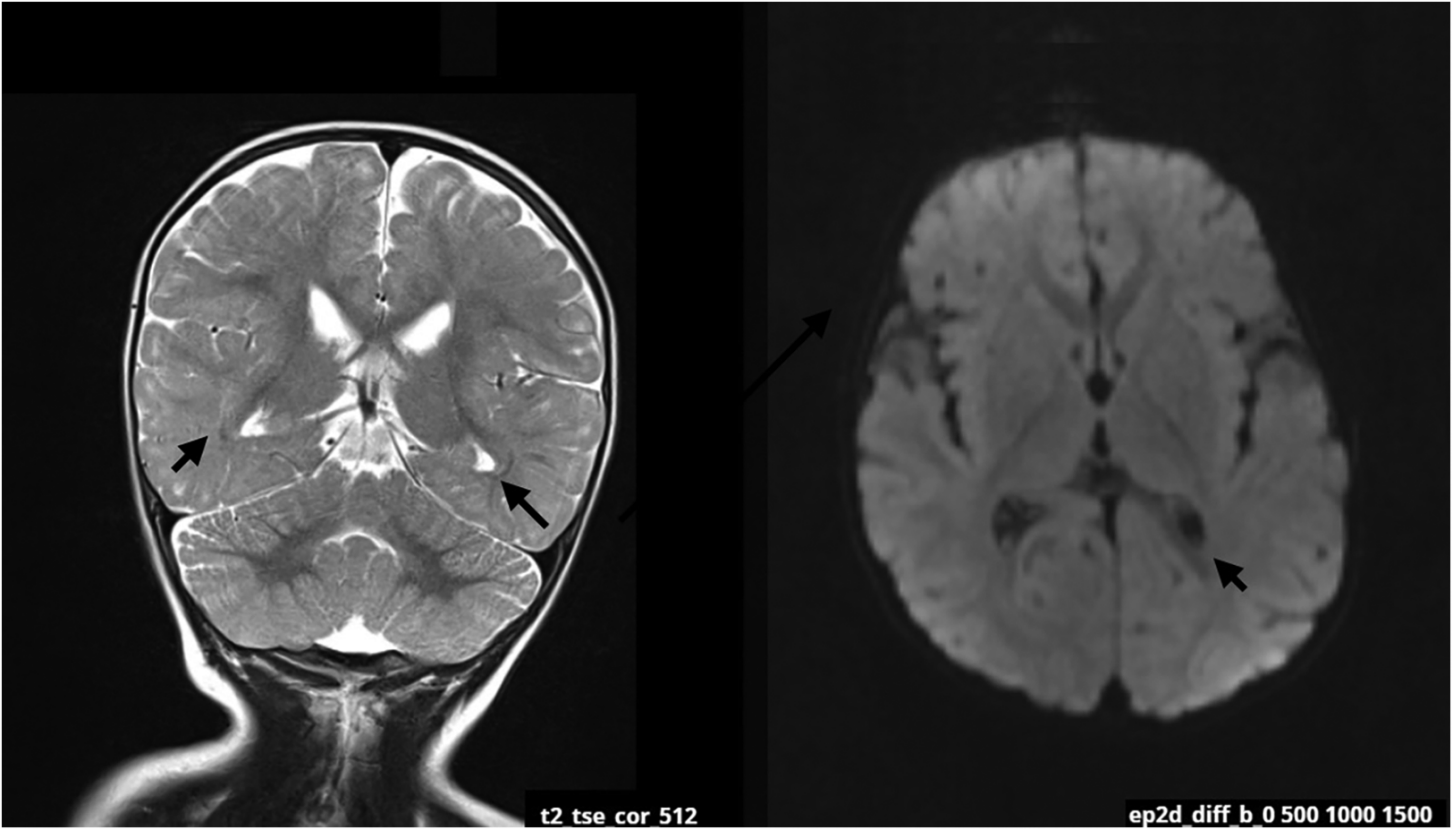
MRI scan at the age of 14 months. The arrows are pointing zones of increased T2/FLAIR signal in the white matter around the occipital horns of the lateral ventricles. The changes may be related to delayed myelination of this region.

At 12 weeks, the mother noticed signs of hypotonia, including the child's difficulty resisting gravity, particularly in the prone position. As developmental delays progressed, particularly the inability to roll from back to front, the parents sought medical help around the child's sixth month of life, marking the beginning of the diagnostic process. Due to the delayed psychomotor development, significant hypotonia, and stereotyped movements, the diagnostic evaluations have been initiated. The patient was qualified for constant physiotherapeutic care and participated in osteopathic treatments. Numerous metabolic tests were performed for metabolic diseases, the results of which were negative. At the age of 14 months, an MRI of the head was performed in the Department of Pediatric Neurology, which revealed discrete zones of increased T2/FLAIR signal in the white matter around the triangles and occipital horns of the lateral ventricles, which may correspond to delayed myelination. No focal changes and cerebellar abnormalities were detected (Figure 1).

**Figure 2:**
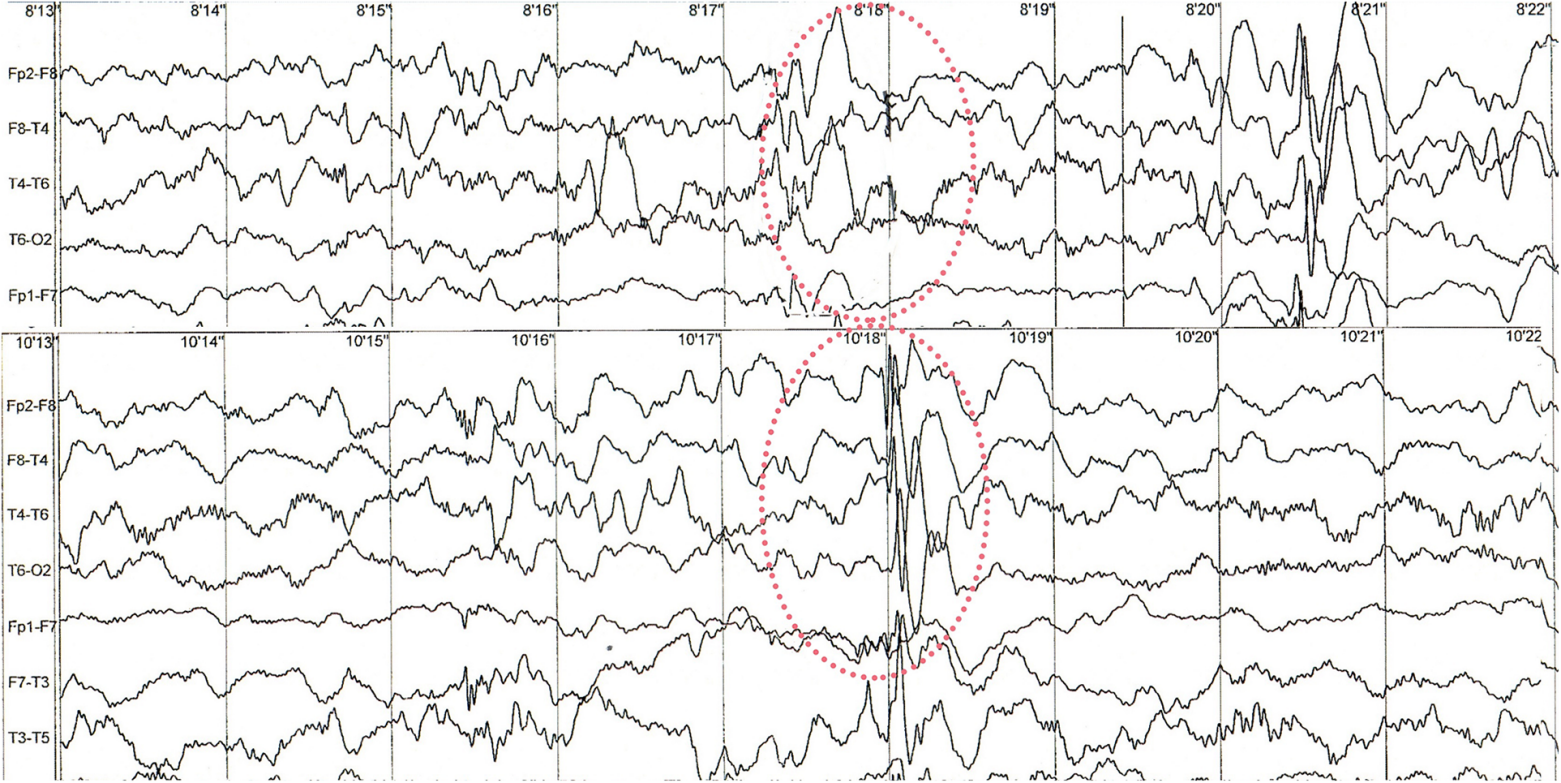
Sample of sleep EEG recording. Red circles are showing abnormal changes in frontotemporal leads. Examples of recorded spike wave complexes with a frequency of 2–2.5 Hz/s and an amplitude of up to 550 uV at 8′18″ and 10′18 with right-sided predominance.

The patient did not have any epileptic seizures while awake and no pharmacological treatment was given. EEG examinations performed during sleep at the age of 14 and 17 months revealed abnormal recordings in the frontotemporal leads with a sharp wave, slow wave and spike wave complexes occurring individually and in groups, with a frequency of 2–2.5 Hz/s and an amplitude of up to 550 uV. Discharges occur bilaterally symmetrically and independently in the right and left hemispheres, with a predominance in the right hemisphere (Figure 2).

**Figure 3:**
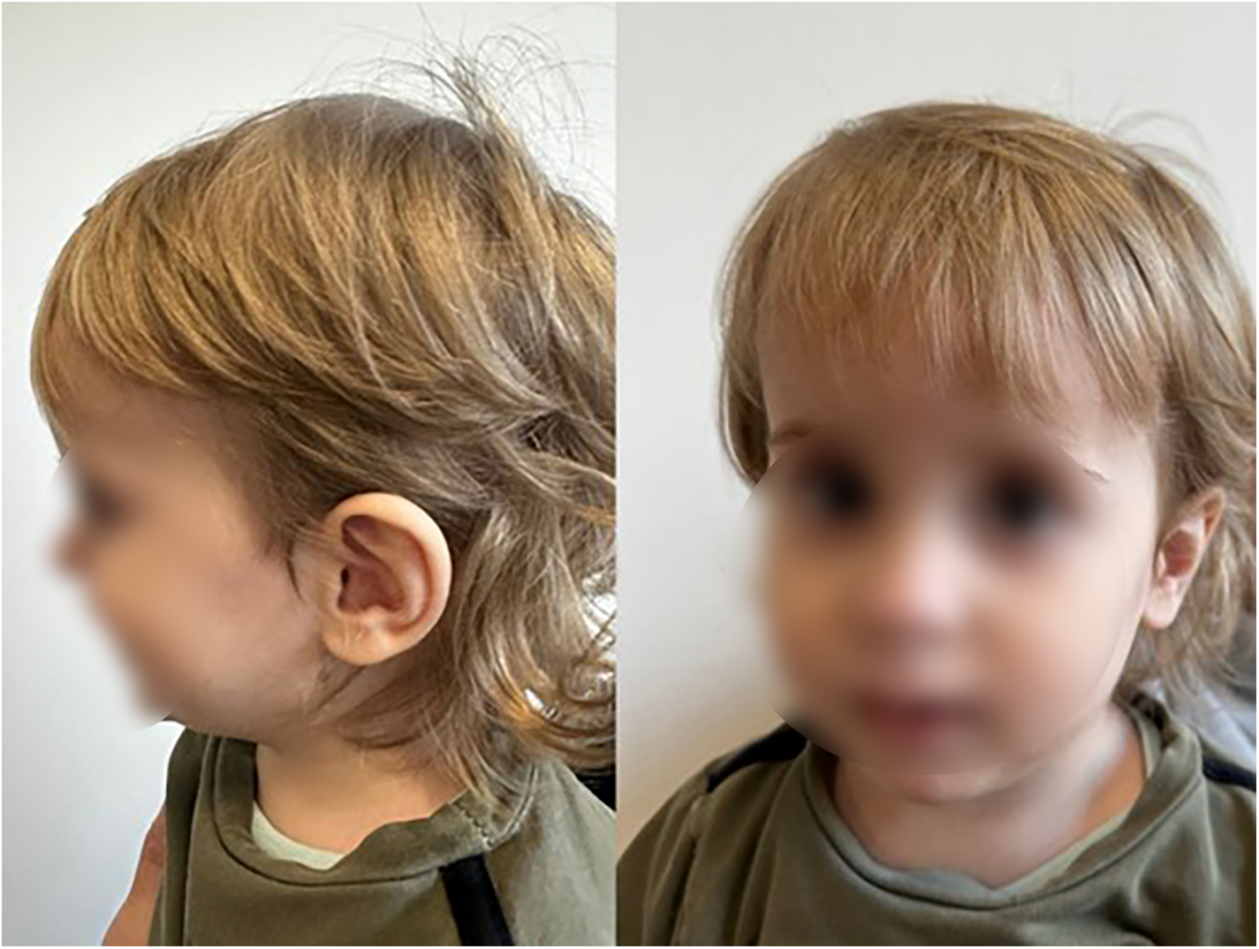
Facial appearance at 2 years and 2 months old with anomalies including a wide and high forehead, a short philtrum, a subtly enlarged earlobe, and an almond-shaped eye (the parents gave written consent to the publication of their son's photos).

Ophthalmological examination revealed hyperopia and astigmatism. The boy is reluctant to wear glasses and takes them off. At the age of 21 months, the selection of glasses was corrected: right eye +0.75/−0.75 × 85, left eye +1.25/−1.25 × 100. At the age of 24 months, patient's body weight is 11.1 kg, OFC 49 cm. The patient has several facial anomalies, including a wide and high forehead, a short philtrum, a subtly enlarged earlobe, and an almond-shaped eye (Figure 3). No changes were observed in the skin, hair or teeth. Sex organs appropriate to age.

### 2.1 Assessment of the patient's psychomotor development

**Figure 4:**
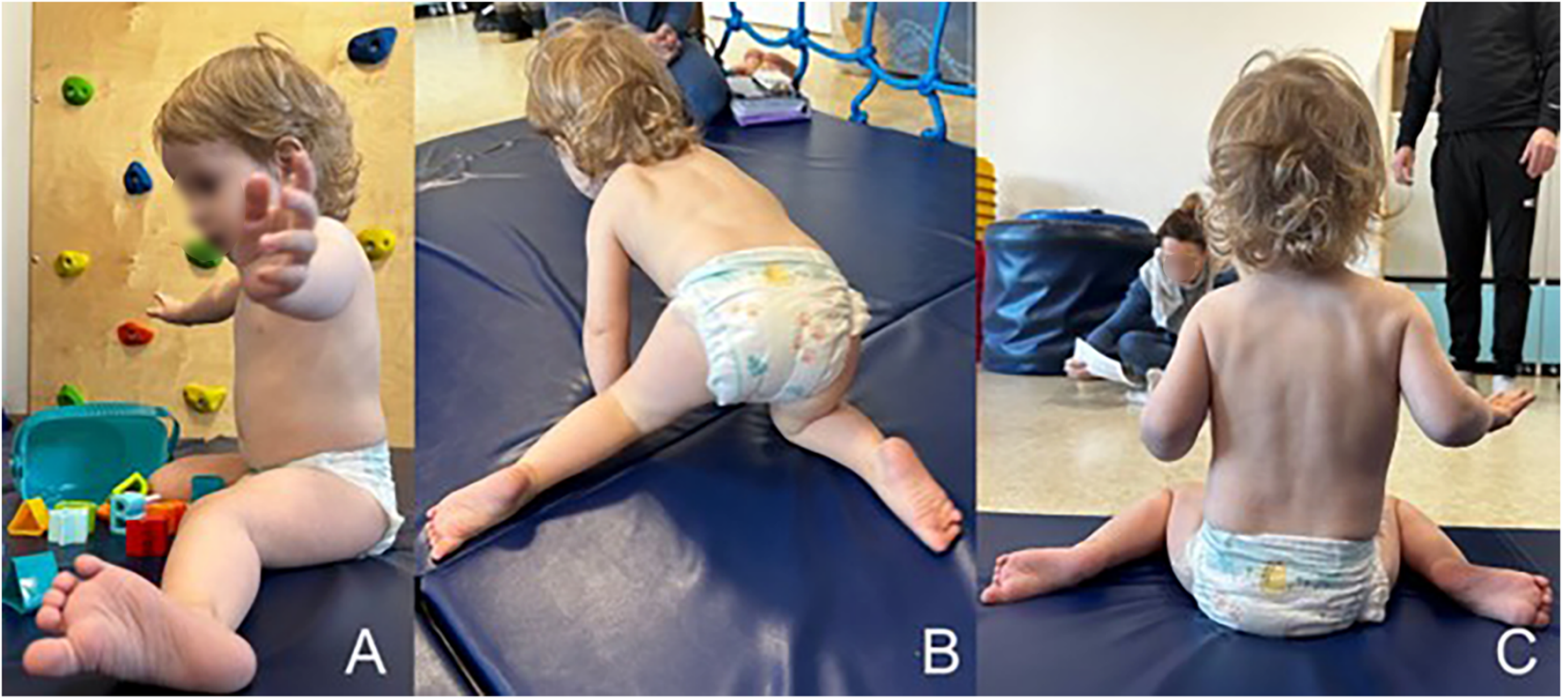
The patient's spontaneous motor skills: **(A)** sitting pattern with a tendency to adduction, internal rotation of the hip joint, valgus of the knee joints and pronated position of the foot, **(B)** a homologous pattern of forward locomotion—“hopping” with asymmetrical positioning of the legs, **(C)** asymmetrical distribution of body weight, with a tendency to shorten the left side of the body.

The patient made his first attempts at independent support in a pronated position at the age of 7 months and achieved an independent sitting position at the 20th month of life. From the age of 22 months, the boy moves forward with a pathological homologous pattern of hopping in all four limbs. During the physiotherapy assessment at 26 months of age, the boy does not stand up kneels on both knees with support on his upper limbs on the parent or on furniture. He grasps with his whole hand with little precision, and he did not demonstrate a tweezers or pincer grip. Decreased muscle tone combined with asymmetry affects the quality of each movement pattern presented by the patient. Due to the current mental needs of the child, who presents patterns bordering on the age of 8 months in the quantitative assessment, the greatest consequences are visible in the position of the lower limbs, which are constantly bent and internally rotated in the hip joints, with intensely expressed valgus of the knee joint and the position of the feet in pronation, more intensely on the left side (Figure 4). During spontaneous assessment and postural tests, patient often hyperextended his knee joints. These tendencies in lower limb control, together with the lack of alternating work, make it much more difficult for the patient to stand upright on his own. The patient responds to painful stimuli adequately, there are no sensory disorders. The pain threshold is normal. In the interview, the parents report avoiding contact with water, especially in the head area. The patient does not say words, he babbles and repeats sequences of syllables, which quantitatively corresponds to the presented motor patterns. The child's behavior indicates features of cognitive development disorder.

## 3 Genetic testing

DNA from the proband and his parents was obtained from peripheral blood leukocytes and extracted with a standard automated protocol. ES (exome sequencing) library preparation for the trio was performed with Twist Exome 2.0 blended and spiked-in with Twist mtDNA Panel (Twist Bioscience, San Francisco, CA, USA). The enriched library was paired-end sequenced (2 × 150 bp) on NovaSeq 6000 (Illumina, San Diego, CA, USA) with final 127,971,107 reads resulting in mean depth of 133.69× (20 × coverage of target bases was 98.4% and 10 × was 98.6%). Reads were aligned to the GRCh38 (hg38).

Data analysis in proband revealed a probably *de novo* heterozygous variant c.2509G>A (annotated to reference NM_020952.6, genomic coordinate 9:070598463-C>T) in TRPM3 gene translated to p.(Val837Met). The variant is absent in gnomAD 4.0.0. browser, evaluated as pathogenic by ACMG criteria (12 points), classified in the ClinVar database as pathogenic/likely pathogenic and has been previously described as disease-causing (6–8, 13–17).

## 4 Discussion

The case presented highlights a 2 years patient, with a pathogenic variant c.2509G>A in the TRPM3 gene, translated as p.Val837Met, contributing to a complex clinical phenotype characterized by developmental delays, neurological abnormalities, and psychomotor impairments. The TRPM3 gene encodes for the transient receptor potential melastatin 3 channel, a member of the TRP superfamily known for its involvement in various cellular functions, including sensory perception and ion transport (2). Several studies in the literature have shed light on the association between mutations in the TRPM3 gene and neurodevelopmental disorders. Dyment at al., for instance, discusses the impact of TRPM3 mutations on neurodevelopment and highlights the variable expressivity of phenotypic features, aligning with the observed clinical heterogeneity in our case (6).

The molecular genetics study provides insights into the underlying genetic mechanisms, emphasizing the role of TRPM3 in neuronal development and its implications for neurocognitive function. The identified mutation in our patient may disrupt the normal function of the TRPM3 channel, leading to altered intracellular signaling and, subsequently, contributing to the observed neurological manifestations (5, 7, 10). Including our case, a total of 18 patients with variant p.Val837Met have been reported to date. Patients often exhibit a spectrum of symptoms affecting various systems, contributing to the complexity of the clinical phenotype (6–9, 16, 17). Neurologically, individuals may present with developmental delays, as observed in the given case, where psychomotor milestones were delayed. The prenatal and perinatal periods were uniformly uncomplicated across all cases presented, including the one described. The infants were delivered in good condition and with birth weights appropriate for their gestational age. Consistent with the case presented, all previously reported patients exhibited intellectual disability ranging from moderate to severe (8). This delay can encompass both gross and fine motor skills, impacting activities such as sitting, standing, and walking. At 26 months of age, the patient had not attained the ability to walk independently, aligning with the clinical profile of previously described patients, in whom this milestone is typically achieved at an average age of 4.1 years (8, 16).

The variable expressivity of symptoms, as highlighted in the literature, underscores the challenges in predicting the specific neurological manifestations associated with TRPM3 mutations (6, 18). Moreover, cognitive and behavioral abnormalities are frequently observed in individuals with TRPM3 pathogenic variants. Cognitive impairments may range from mild to severe, affecting language development, learning abilities, and overall intellectual functioning (19). The literature indicates that the impact of TRPM3 dysfunction on neuronal development could contribute to these cognitive manifestations. Additionally, behavioral concerns, including anxiety or stereotypical movements, may occur, as observed in our patient (7).

The literature reveals a diverse array of symptoms associated with TRPM3-related disorders, highlighting the complexity and variability of this neurodevelopmental condition. Patients frequently present with hypotonia, which contributes to delays in both gross and fine motor skills, as observed in previous studies (6, 8, 16). These motor deficits may be accompanied by impaired coordination and balance, further affecting daily living activities. Seizures have been observed in approximately half of the affected individuals, underscoring the range of neurological manifestations and their potential impact on overall neurocognitive function (9). Among those affected, they were reported as stable or well-controlled, regardless of medication. This aligns with our case, where the boy has not experienced any seizures despite abnormal EEG results (8). However, since the average age of onset for epileptic events range from 9 months to 7 years, the possibility of such events developing in the 2-year-old patient cannot be excluded (7).

Cognitive impairments represent a significant aspect of TRPM3-related disorders, affecting various domains of intellectual functioning. Language development may be notably delayed, with some individuals exhibiting limited speech or communication skills. While few individuals achieved some level of verbal communication, only 3 of them developed the ability to combine words and sufficient oral language skills (8, 16). The average age at which the first words were spoken was 3.7 years (8). Our patient has not yet exhibited the ability to speak; however, it is important to consider his relatively young age compared to the known cases. Additionally, intellectual disability ranging from mild to severe has been reported, emphasizing the wide-ranging impact on cognitive abilities (18).

Behavioral abnormalities are common in patients with TRPM3 mutations, adding layer of complexity to the clinical presentation. Anxiety, stereotypical movements, and social difficulties are frequently observed, contributing to challenges in interpersonal relationships and adaptive behaviors (17). Literature discusses the intricate interplay between the neurological and behavioral aspects of TRPM3-related disorders, further highlighting the need for a comprehensive understanding of the condition (7). As mentioned in the presented case, visual disturbances, including astigmatism and hypermetropia, underscore the multisystem nature of TRPM3. These ophthalmological findings may add to the clinical spectrum and require specific attention in managing affected individuals. Despite the literature noting an elevated pain threshold and diminished sensitivity to heat, these characteristics are not frequently encountered within the spectrum of this disorder and were not observed in our patient (8, 16). An affinity for water and heightened tactile sensitivity have not been previously reported and represent distinctive features.

In summary, the clinical spectrum of conditions associated with TRPM3 encompasses a wide range of neurological, cognitive, and behavioral symptoms. Understanding these diverse manifestations is crucial for early diagnosis, comprehensive patient care, and ongoing research efforts aimed at elucidating the underlying pathophysiological mechanisms of this complex genetic condition.

## 5 Management considerations

Accurate and timely diagnosis is essential for appropriate management. The diagnostic process involves a combination of genetic testing, neuroimaging, and clinical assessments. Genetic testing, such as whole-exome sequencing, can identify pathogenic variants in the TRPM3 gene. Neuroimaging, including brain MRI, may reveal structural abnormalities or delayed myelination, as seen in the presented case. Clinical evaluations encompass a comprehensive assessment of developmental milestones, cognitive abilities, and behavioral patterns to construct a holistic understanding of the patient's condition. Collaboration between geneticists, neurologists, therapists, and other specialists is paramount to navigating the diagnostic and therapeutic challenges associated with TRPM3-related disorders (8, 16, 17, 19).

Currently, there is no direct causal treatment available for patients with TRPM3 gene variants. However, promising literature reports suggest potential benefits from the use of the antiepileptic drug primidone in these patients. The increased basal channel activity and the associated elevated calcium levels observed in all characterized disease-related TRPM3 variants can be inhibited by high doses of primidone, which has been identified as a direct antagonist of TRPM3 *in vitro* and in animal models (9, 20). The introduction of this treatment in children diagnosed with neurodevelopmental disorders caused by TRPM3 variants has led to significant improvements, including enhanced EEG results, a reduction in seizure frequency up to complete cessation, and improved cognitive abilities, all without observed side effects. This was also noted in patients with the Val837Met variant. Although the drug does not exhibit selectivity for TRPM3 receptors, making it challenging to entirely exclude the possibility that the therapeutic effect is also due to the action of phenobarbital (a metabolite of primidone) on γ-aminobutyric acid type A (GABA-A) receptors, these findings support the use of primidone as a therapeutic option for patients with TRPM3-related disorders (21).

## 6 Conclusions

Our case provides a clinical context for the discussion of TRPM3-related disorders. The literature reviewed emphasizes the importance of genetic testing and comprehensive clinical assessments in understanding the spectrum of phenotypic variability associated with TRPM3 mutations. The symptomatic manifestations of TRPM3-related disorders encompass a broad spectrum of neurological, cognitive, and behavioral features. Understanding the diverse clinical presentation is crucial for early identification and intervention, emphasizing the importance of a multidisciplinary diagnostic approach. Further research is needed to elucidate the intricate mechanisms underlying these symptoms and to develop targeted therapeutic strategies for individuals affected by TRPM3 mutations.

## Data Availability

The original contributions presented in the study are included in the article/Supplementary Material, further inquiries can be directed to the corresponding author.
